# Cough management: a practical approach

**DOI:** 10.1186/1745-9974-7-7

**Published:** 2011-10-10

**Authors:** Francesco De Blasio, Johann C Virchow, Mario Polverino, Alessandro Zanasi, Panagiotis K Behrakis, Gunsely Kilinç, Rossella Balsamo, Gianluca De Danieli, Luigi Lanata

**Affiliations:** 1 Department of Respiratory Medicine and Pulmonary Rehabilitation, Clinic Center, Private Hospital, Naples, Italy; 2 Department of Pneumology and Intensive Care Medicine, University of Rostock, Germany; 3 Department of Medicine and Medical Specialities, Azienda Sanitaria Locale (ASL) Salerno, Italy; 4 Centre for Study and Treatment of Cough Respiratory Medicine, S. Orsola, Malpighi Hospital Bologna, Italy; 5 Department of Experimental Physiology, University of Athens, Greece; 6 Department of Chest Disease, Cerrahpasa Faculty of Medicine, University of Istanbul, Turkey; 7 Medical Department, Dompé S.P.A, via San Martino 12, Milan, Italy

**Keywords:** cough, cough reflex, acute, chronic, diagnosis, treatment

## Abstract

Cough is one of the most common symptoms for which patients seek medical attention from primary care physicians and pulmonologists. Cough is an important defensive reflex that enhances the clearance of secretions and particles from the airways and protects the lower airways from the aspiration of foreign materials. Therapeutic suppression of cough may be either disease-specific or symptom related. The potential benefits of an early treatment of cough could include the prevention of the vicious cycle of cough. There has been a long tradition in acute cough, which is frequently due to upper respiratory tract infections, to use symptom-related anti-tussives. Suppression of cough (during chronic cough) may be achieved by disease-specific therapies, but in many patients it is often necessary to use symptomatic anti-tussives, too. According to the current guidelines of the American College of Chest Physician on "Cough Suppressants and Pharmacologic Protussive Therapy" and additional clinical trials on the most frequent anti-tussive drugs, it should be possible to diagnose and treat cough successfully in a majority of cases. Among drugs used for the symptomatic treatment of cough, peripherally acting anti-tussives such as levodropropizine and moguisteine show the highest level of benefit and should be recommended especially in children. By improving our understanding of the specific effects of these anti-tussive agents, the therapeutic use of these drugs may be refined. The present review provides a summary of the most clinically relevant anti-tussive drugs in addition to their potential mechanism of action.

## Introduction

In addition to being an airway defence mechanism, coughing is a very common symptom observed in many diseases other than those affecting the respiratory system. To recognize its cause is not always an easy task. Where possible, the clinician should avoid treatment based on symptoms only which often only serves the purpose to reassure the patient or the parents (in the case of a paediatric patient). On the other hand it is worth mentioning that internal medicine physicians are frequently overwhelmed by requests for help by patients who report coughing, alone or together with other non-specific symptoms such as malaise, pharyngodynia, and a mild temperature. In such cases, treatment of symptoms alone appears justified as a therapeutic approach. However, it must be emphasized that a high level of suspicion needs to be maintained, especially when coughing persists which would require a thorough investigation of other possible causes.

This review summarises the effectiveness of symptomatic cough remedies including two specific drugs (levodropropizine and moguisteine) which have been tested in the symptomatic treatment of cough, and have received Grade A evidence in the treatment of cough due to either acute or chronic bronchitis. In addition we identify missing pieces of evidence regarding the efficacy of symptomatic cough treatments as well as associated side effects. Moreover, clear treatment algorhythms still need to be established for acute and chronic cough

## Methods

A thorough systematic literature search was conducted in the main international search databases (Pubmed, Embase, Biosis) of all articles (both original clinical trials and reviews) published in the period from 1950 up to now. For this search, all keywords related to cough (acute, sub acute and chronic), cough mechanism and pathogenesis, cough treatment (cough suppressants, anti-tussives and other drugs with anti-tussive activity) were used.

Authors' recommendations were based on this clinical evidence and on available guidelines for clinical practice

### Definition and causes of acute, sub acute and chronic cough

**Acute cough **is rather arbitrarily referred to as a cough lasting for a maximum of 3 weeks. In the majority of patients, it is caused by upper respiratory tract infections (URTI), acute bronchitis or tracheo-bronchitis due to bacterial or more frequently viral infections [1]. It has been estimated that only few patients with URTI-induced cough seek medical attention. Acute cough due to such infections is usually self-limited and subsides within one to two weeks along with the clearing of the infection.

There are no targets or reliable measures to predict the duration of a cough at its onset (i.e., resolution within 3 weeks). Neither is it possible to predict which cough will persist into the sub acute or chronic stage. The issue is further complicated by the fact that effective therapy can abort or abbreviate the duration of a cough, whereas failure to institute effective therapy can convert what might have been an acute cough into a sub acute or chronic one. Furthermore, recurrent acute episodes of cough can be a manifestation of an undiagnosed chronic disease (e.g., asthma). Nevertheless, keeping these caveats in mind, a relatively ''standard'' diagnostic and therapeutic approach based on the duration of the cough has proved useful [2-4].

**Sub acute cough **has been defined as a cough lasting for 3-8 weeks. Following specific infections (e.g., *M. pneumoniae*), an increase in bronchial hyper-responsiveness may persist, which can cause or maintain sub acute cough that can remain bothersome for a period of weeks even after the inciting infection has completely resolved. Post-infectious airway hyperresponsiveness resulting in a sub acute cough has been scarcely studied. Randomised, controlled trials to prevent and/or treat this condition are missing. Although inhaled corticosteroids or leukotriene receptor antagonists are frequently prescribed for this condition, there is no controlled scientific evidence to support their use, which is self-limited in many cases. Further causes of sub acute cough include *B. pertussis*, where coughing persists with disabling paroxysms, despite resolution of the infection. While the rate of persons vaccinated decreases, pertussis-induced cough becomes more frequent in several countries [5]. Recent pertussis infection should be ruled out in children and adults with sub acute cough irrespective of any prior vaccination. Cough as a result of a *B. pertussis *infection usually leads to paroxysmal episodes of coughing with a characteristic inspiratory whoop, especially in children. However, this can be absent, especially in adults. Non-infectious causes of sub acute cough include gastroesophageal reflux, aspiration and bronchial asthma, which is a likely diagnosis when cutaneous sensitisations to seasonal allergens can be shown in an allergen skin test or if symptoms occur following exposure to environmental allergens or pollutants. Subclinical congestive heart failure can be a cause of acute and sub acute cough, especially during periods of fluid overload. Rare cases of sub acute cough include pulmonary sequestration, and very occasionally Tourette's syndrome, which can manifest itself solely as paroxysmal coughing episodes

### Differential diagnosis of acute and sub acute cough

**Figure 1:**
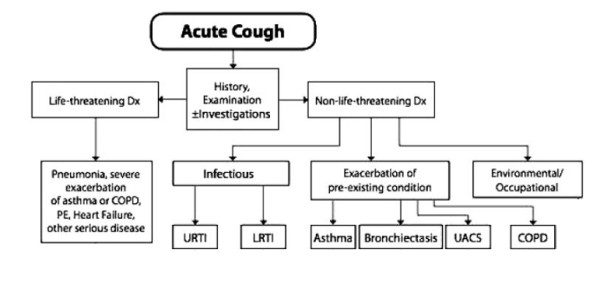
**The acute cough algorithm for the management of patients aged ≥15 years with cough lasting < 3 weeks**. PE = pulmonary embolism; Dx = diagnosis; Rx = treatment; URTI = upper respiratory tract infection; LRTI = lower-respiratory tract infection. Taken from Ref [61] with permission from the publisher.

**Figure 2:**
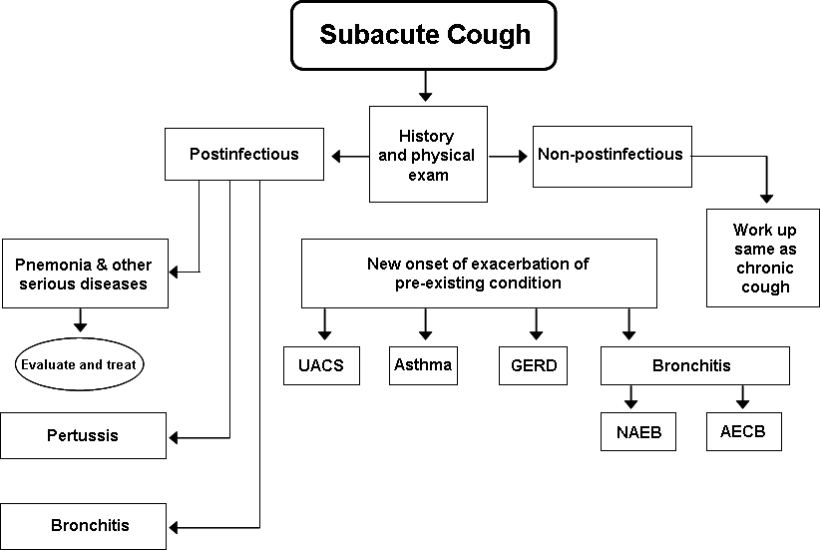
**Sub acute cough algorithm for the management of patients aged > 15 years with cough lasting 3 to 8 weeks**. See the legend of Figure 1 for other section references. Taken from Ref [61] with permission from the publisher.

The differential diagnosis of acute and sub acute cough is wide ranging and includes a plethora of diseases. Chronic cough is most frequently related to the chronic inhalation of cigarette smoke by either active or passive smoking [6]. The diagnostic challenge for clinician faced with acute or sub-acute chough is the identification of benign, self-limited episodes of mostly infection associated cough versus severe, potentially life-threatening diseases as the underlying cause of the presenting cough. Exposure to particulate matter has also been identified as a source of cough [7]. However, most cases of acute and sub acute cough are caused by broncho-pulmonary infections from various organisms [8]. There is little doubt that environmental and infectious mechanisms can synergistically contribute to the pathogenesis as well as the severity and duration of the cough but this has not been fully evaluated. The major challenge for the clinician still remains to be the early identification of severe underlying diseases, such as bronchial carcinoma or tuberculosis in patients with cough of recent onset that does not yet fulfil the criteria of a chronic cough. The general approach to the treatment of a patient with any cough begins with a search for the cause of any acute and/or sub acute cough (Figure 1 and 2). This involves differentiation into relatively benign but also potentially life-threatening causes. A detailed history is key to the identification of the underlying cause and any subsequent decision if treatment for cough or its underlying condition is necessary. The onset of cough can provide initial clues as to its origin. Abrupt onset can be related to aspiration, especially in small children and elderly persons. Signs and symptoms of an upper respiratory tract infection point to the most common and usually benign reasons for acute or sub acute cough. However, they can precede severe pneumonia and therefore occasionally require close observation. A history or signs and symptoms of gastroesophageal reflux can be associated with intermittent cough. A detailed history of recent medication can reveal ACE-inhibitors and beta blockers as causes of cough [9]. This usually occurs within the first few days after initiation of treatment, but can occur even after prolonged periods of previous therapy. A detailed smoking history including the number of pack years as well as the age of onset of smoking is mandatory in the workup of any cough. Exposures at the workplace to noxious and/or sensitising agents are often overlooked as a cause of cough or an occupational asthma presenting as cough. Troublesome nocturnal coughing episodes which may include vomiting should prompt investigation into recently acquired pertussis, gastroesophageal reflux and more frequently asthma, especially in children. In addition, in many patients, especially young children, cough is often the first and only symptom of asthma. A detailed history into potentially aggravating factors as well as the nature of the cough, namely productive or non-productive forms of sputum may be helpful.

The clinical examination of a patient with cough includes the nose, for obstruction and/or discharge as well as the oropharynx which should be closely inspected for direct or indirect signs of postnasal drip or other abnormalities. The ear, innervated by the vagal nerve, can also be a cause of cough. Therefore, the external acoustic meatus should also be examined to exclude cerumen or other foreign bodies. A detailed examination of the chest, including the heart, is mandatory but goes beyond the scope of this article. According to most guidelines, a chest radiograph in the anterior and lateral view is warranted in any unexplained cough that persists for more than 2 weeks.

In the case of sputum production, this should be analysed for the approximate quantity and colour, which can suggest bacterial growth. However, in most cases of uncomplicated URTI, sputum bacteriology is not needed and should be reserved for severe or complicated cases such as in the setting of immune-suppression or co-morbid lung disease warranting antibiotic therapy. Sputum cytology is an underused diagnostic tool which should be employed in any patient with a smoking history and an unexplained cough. The diagnosis of psychogenic cough, although probably not uncommon, needs to remain a diagnosis of exclusion.

With cough being one of the most frequent symptoms of patients seeking medical advice from general practitioners and specialists, the difficult task of physicians caring for patients with cough is to identify severe causes such as neoplastic disease, severe infections (e.g., tuberculosis, etc.) and inflammatory conditions (e.g., Wegener's granulomatosis, etc.) without subjecting every patient with benign, self-limiting cough to extensive diagnostic procedures.

### Chronic and persisten cough

In 1977, R. S. Irwin reviewed the most common causes of persistent and chronic cough [10]. In that paper, he postulated that due to the fact that the numbers of anatomic locations for afferent cough receptors were small the number of diseases or conditions that could stimulate these sites and result in chronic or persistent cough should be equally limited. Subsequent descriptive studies in the literature that looked at patient populations seeking medical attention for a primary complaint of cough have in fact reinforced this spectrum of conditions as initially postulated. Only in a small proportion of patients with chronic cough which is either due to cigarette smoking or the use of an ACE inhibitor the cause of cough could be determined [11-13]. On the other hand, in the vast majority of the remaining patients, the following three dominant etiologies have emerged to explain the causes of chronic cough: upper airway cough syndrome (UACS) due to a variety of rhinosinus conditions, which was previously referred to as postnasal drip syndrome (PNDS); asthma; and GERD [11-15]. In four prospective studies from the Western World, this triad of diagnoses was so ubiquitous that in 92 to 100% of patients who were nonsmokers, and who were not using an ACE inhibitor, and who had normal chest roentgenogram findings, the presence of one, two, or even all three of these conditions proved to be the etiologic explanation for chronic cough [11,15-17]. Even in the less industrialized areas of the world (i.e. where tuberculosis is endemic, and was an important consideration as a cause of chronic cough), UACS, asthma, nonasthmatic eosinophilic bronchitis (NAEB), and GERD are still the most common causes seen.

**Figure 3:**
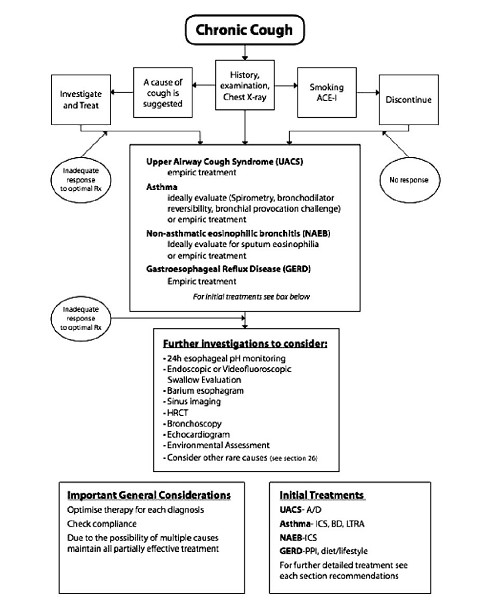
**Chronic cough algorithm for the management of cough ≥15 years of age with cough lasting > 8 weeks**. ACE-I; ACE-inhibitor; BD = Bronchodilator; LTRA = Leukotrienes receptor antagonist; PPI = Proton Pump Inhibitor. Taken from Ref [61] with permission from the publisher.

It should to be clearly recognized that each of these entities may present only as cough with no other associated clinical findings (i.e., "silent PNDS" [now termed UACS], "cough variant asthma, " and "silent GERD") [13,18,19]. It is also important to note that the medical history is of little value as the patient's description of his or her cough in terms of its character or timing, or the presence or absence of sputum production is of little diagnostic value [15,17]. Even in the presence of significant hypersecretion, a nonsmoking patient who is not receiving an ACE inhibitor and who has a normal chest roentgenogram will usually turn out to be coughing due to UACS, asthma, GERD, or some combination of these diagnoses [17]. Nevertheless, the medical history is important to rule out ACE inhibitor therapy, current as well as a former smoking, or exposure to tuberculosis or certain endemic fungal diseases. In addition a previous history of cancer, tuberculosis, or AIDS, or other systemic symptoms of fever, sweats, or weight loss require consideration. An algorithm for the management of chronic cough is shown in Figure 3.

However, it still remains important to recognize that there are a number of other conditions, although on average much less common, that may account for an important percentage of cases of chronic cough. For example, NAEB, which is a disorder that is characterized by cough, eosinophilic infiltration of the bronchial tree, normal spirometry findings, a lack of bronchial hyperresponsiveness, and a resolution of both cough and eosinophilia with steroid treatment, [20-23] has been reported to have a prevalence as an etiology of chronic cough from as low as 13% to as high as 33% in a number of studies [16,23-26]. Until today, only a few large studies were able to define the etiology of a chronic cough in up to 100% of cases without reporting a single case of NAEB [11-14]. Nevertheless, a diagnosis of NAEB should be considered early in the diagnostic evaluation as it can be reliably determined by induced sputum stained for eosinophils, and by its rapid response to (inhaled) corticosteroid therapy.

While one series [14] of patients with chronic cough (performed in the US) described a significant number of patients with "postinfectious" cough, other series [11,13-16] were able to reach a high diagnostic yield without using this category. The implication is that most of the cases of post infectious cough had UACS as their persistent path-physiology, transient bronchial hyperresponsiveness, or prolonged airway inflammation that resolved as diagnostic/therapeutic studies were being pursued. Similarly, patients with bronchiectasis from a variety of causes, endobronchial abnormalities (e.g., tumors, tuberculosis, sarcoidosis, or retained sutures), isolated suppurative lower airway infection, congestive heart failure, thyroid disease, habitual or psychogenic cough, neuromuscular disorders, or a mediastinal mass, will occasionally present with chronic cough as the major manifestation.

In conclusion, the most common causes of chronic cough are UACS due to a variety of rhinosinus conditions, asthma, and GERD. Each of these diagnoses may be present alone or in combination and may be clinically silent apart from the cough itself. While there are a number of other conditions that can result in chronic cough, in the absence of evidence suggesting the presence of one of these other disorders, an approach strongly focused on initially detecting the presence of UACS, asthma, or GERD, alone or in combination, is likely to have a far higher yield than routinely searching for relatively uncommon or obscure diagnoses. The one exception to this is that NAEB may be more important than has often been recognized, is relatively easy to diagnose with the appropriate laboratories workup, and therefore should also be considered early in the diagnostic evaluation.

### Anti-Tussive Drugs

#### Central Antitussives

Currently available cough suppressants include centrally acting drugs (opioids and non opioids) and peripherally acting anti-tussives.

Opioids, such as morphine and codeine, [27,28] are believed to inhibit cough primarily by their effect on the cough center; opiate anti-tussives have a greater adverse side effect profile. Because of the potential for abuse and addiction with opioids, nonopioid anti-tussives (e.g., dextromethorphan) are preferred in the treatment of acute cough. They are widely available without prescription and thus classified as over-the-counter (OTC) drugs. A meta-analysis of five studies with Dextrhomethorphan and Codeine in adults concluded that these central anti-tussives that these drugs have demonstrated have marginally superior to placebo [29]. Table 1, 2

**Table 1:** Clinical Studies with Codeine

Study	Sample Size	Design	Disease	Results
**Eccles R, et al**. Lack of effect of codeine in the treat- ment of cough associated with acute upper respiratory tract infection. Journal of Clinical Pharmacy and Therapeutics 1992;17(3):175-80.	81 adults	Not reported	URTI's	Codeine was no more effective than placebo either as a single dose or in a total daily dose of 120 mg, reported on a five-point cough severity score (P > 0.2).
**Freestone C et al**. Assessment of the antitussive efficacy of codeine in cough associated with common cold. Journal of Pharmacy and Pharmacology 1997;49:1045-9.	82 adults	A double-blind, stratified, placebo-controlled, parallel-group,	URTI's	The results demonstrate that codeine is no more effective than placebo in reducing cough associated with acute URTI, as measured by CSPLs, cough frequency or subjective symptom scores

**Table 2:** Clinical Studies with Dextromethorphan

Study	Sample Size	Design	Disease	Results
**Lee PCL et al**. Antitussive efficacy dextromethorphan in cough associated with acute upper respiratory in- fection. *Journal of Pharmacy and Pharmacology *2000;**52**:1137-42.	44 adults	A double-blind, stratified, randomized and parallel group design	URTI's	This study provides very little if any support for clinically significant antitussive activity of a single 30 mg dose of dextromethorphan in patients with cough associated with URTI's
**Parvez L, et al **Evaluation of antitussive agents in man. *Pulmonary Pharmacology* 1996;**9**(5-6):299-308.	451 adults	Review of three different studies randomized, double blind, placebo controlled	URTI's	The results establish the sensitivity and robustness of the cough quantization methodology in the objective evaluation of cough treatments
**Pavesi L et al**. Application and validation of a computerized cough acquisition system for objective monitoring of acute cough: a meta-analysis. Chest 001;120:1121-8.	710 adults	Six studies used for the meta-analysis were randomized, double-blind, parallel-group, single-dose, placebo-controlled studies with a 3-h postdose cough evaluation period	URTI's	The results of a meta-analysis show that the antitussive effect of a single dose of dextromethorphan hydrobromide, 30 mg, has been established.

#### OTC Medicines

Self-prescribe OTC preparations which include combinations of antihistamines, decongestants, cough suppressants and expectorants are frequently used.

A critical analysis of a Cochrane review suggests that the effectiveness of OTC medicines in acute cough is weak [30]. These results, however, require a careful interpretation because of differences in patient characteristics and the quality of the studies examined. Accordingly, some trials in the literature have generated conflicting results which question their clinical relevance.

This Cochrane review of the literature [30] has documented that, at least in adults, studies that compared cough suppressants to placebo produced variable results. Two trials have compared the expectorant guaifenesin to placebo [31,32], but only one study showed a significant advantage for the expectorant [33]. Another study showed that a mucolytic can reduce the frequency and the intensity of cough [31]. Two trials have examined the combination of an antihistamine and a decongestant with equivocal findings [33,34]. Three other studies compared other combinations of drugs with placebo and showed some benefits in reducing cough [35-37]. Three trials have concluded that antihistamines are not more effective than placebo in relieving cough [33,38,39]. In children, cough suppressants (two studies, one with dextromethorphan [40] and another study with dextromethorphan plus codeine [41], antihistamines [42,43] (two studies), antihistamine- decongestant combination [44,45] (two studies) and bronchodilator-cough suppressant combination (one study) were not more effective than placebo [40].

#### Peripheral Antitussive

Regarding peripherally acting anti-tussives, levodropropizine, which is an orally-administered non-opiod agent whose peripheral anti-tussive action may result from its modulation of sensory neuropeptide levels within the respiratory tract [46] In clinical trials conducted in adults, levodropropizine was compared in a double blind studies with placebo, morclofone, cloperastine dextromethorphan and codeine. Table 3

**Table 3:** Clinical Studies with Levodropropizine vs Central antitussives in adults

Study	Sample Size	Design	Disease	Results
**Allegra et al**. Arneim. Forsch./Drug Res., vol. 38 (II) 8: 1163-6, 1988.	174 adults	The studies have been conducted as follow: 1-2 Studies: vs Placebo 3-4 Studies: vs Morclofone 1% 5-6 studies: Cloperastine drops 2%	Bronchitis	- LDP antitussive action resulted in being higher than placebo and morclofone and similar to cloperastin. - LDP showed effective in about 80% patients: in responders the cough frequency was reduced by 33-51%.
**Catena. et al**. Pulmonary Pharmacology & Therapeutics 1997; 10: 89-96.	209 adults	Double blind randomized vs Dextromethorphan	Non productive cough	- The results bear out the effectiveness of LDP as an antitussive comparable with dextromethorphan - The results support a less incidence of somnolence and nightly awakenings in the LDP's group
**Luporini G. et al **Eur Respir J 1998; 12: 97-101	140 adults	Double blind randomized vs Dihydrocodeine	Lung Tumor	- The antitussive effect of LDP was comparable with the reference drug, Dihydrocodeine. LDP induced significantly less somnolence compared to Dihydrocodeine.

The anti-tussive activity and therapeutic efficacy of the levodropropizine were shown to be greater than placebo and morclofone and similar to cloperastine [47]. Levodropropizine was also compared to dextromethorphan in a double blind randomized study in adults. The purpose of this study was to confirm levodropropizine's efficacy and tolerability and the absence of effects on CNS. The anti-tussive activity of levodropropizine was found to be comparable with dextromethorphan. Subjects in the levodropropizine group also reported less somnolence and nocturnal awakenings [48].

Levodropropizine was also studied in cough due to advanced cancer [49] and interstitial lung disorders [50]. Collectively, these studies have confirmed its anti-tussive effect and have suggested a favourable benefit/risk profile.

Moreover, several clinical trials have demonstrated the efficacy and tolerability of levodropropizine in paediatric patients not only in open label studies [51-53], but also when compared with central antitussive drugs [54,55]. Table 4

**Table 4:** Clinical Studies with Levodropropizine vs Central antitussives in Children

Study	Sample Size	Design	Disease	Results
**Banderali et. al **J Int Med Res 1995 May-jun;23(3): 175-83	254 children aged between 2 and 14 yrs	Double blind randomized Dropropizine vs Levodropropizine	Non-Productive Cough	There were statistically significant decreases in the frequency of coughing spells and nocturnal awakenings after both LDP and dropropizine treatments with no statistical difference between both group. Somnolence was twice as frequent in the dropropizine group (10.3% vs 5.3%) and the difference is clinically relevant, though not statistically significant.
**Dong Soo Kim et al**. Diagnosis and Treatment Vol 22. Num 9. 2002	77 children aged 2 and 3 years	Double blind randomized LDP vs Dextromethorphan	Bronchitis	The results show the antitussive effectiveness of LDP and point out a more favourable benefit/risk profile when compared with dextromethorphan.
**Fiocchi A et al**. Ped Med Chir 1989	70 children, age ranged between 2 months and 14 years	Open Label	Respiratory tract disease	- The treatment was effective on 69/70 children. No child showed a worsening in the cough after 24 hours treatment.
**Tamburrano et al**. Terapie essenziali in clinica 1989; 3-7	180 children aged between 5 months and 12 years	Open Label	Respiratory tract disease	- The results of the present study prove that the treatment with LDP in children is excellently tolerated and clinically active
**Banderali et al **Study LPD 0191. Data on file Unplublished	325 children aged between 2 and 14 years	Open label	Non-productive cough	- This study proved the a favourable therapeutic results with limited risk of inefficacy, with the subsequent improvement in the patient's and parents' quality of life, and with remarkably limited risk of intolerance, especially in terms of daytime somnolence.

In one study the efficacy and tolerability of levodropropizine was compared with dropropizine in the management of non-productive cough in paediatric patients. In this study the anti-tussive effects of levodropropizine were similar to dropropizine, but it caused less daytime somnolence [54]. In another study in children with bronchitis levodropropizine provided anti-tussive efficacy with a more favourable risk/benefit ratio when compared with dextromethorphan [55].

### Treatment

#### Acute and sub acute cough

Satisfactory control of acute and sub acute cough is not achieved in many patients resulting in substantial morbidity, decrease in quality of life and loss of productivity. Therapeutic interventions primarily aim at removing the underlying cause of cough. Irrespective of this, the treatment of cough often requires symptom related approaches. Ideally, treatment of the underlying cause(s) of cough with specific treatments should eliminate cough. This approach may not be successful if no cause can be established, if treatment of the underlying disease has a delayed onset of action or if this treatment fails. Empiric treatment with anti-tussive agents is often needed in particular when associated with deterioration in the quality of life. A concept that is worth highlighting is the importance of treating cough to avoid the development of persistent cough. The potential benefits of initiating treatment early could be in preventing the vicious cycle of cough perpetuating cough and decreasing the infectious spread of viruses [56]. The prevention of the vicious cycle of cough could avoid many related complications, such as fatigue, sleep deprivation, hoarseness, musculoskeletal pain, sweating, and urinary incontinence [56].

There may be two independent mechanisms involved. The acute phase of the cough could be caused by a respiratory virus or by an episode of gastroesophageal reflux disease (GERD) through direct stimulation of cough receptors. The inducing agent may also be involved in the process of sensitization that may contribute to a more persistent cough. The initiating event may have disappeared, leaving a persistent cough with or without apparent cause. This could result in an "idiopathic" cough, or in a cough that does not respond to specific therapies of the associated cause(s). According to this hypothesis, a non-specific approach to cough suppression is always necessary [57].

While earlier studies supported their use in acute cough more recent data suggests ineffectiveness of codeine in the suppression of cough in the setting of common colds [58].

The Food and Drug Administration (FDA) issued a warning for parents and health workers against the use of OTC products for cough and common colds in infants and children under 2 years of age because of serious side effects and the potential danger to life that may arise as a result of their use in children [59,60]. It should be pointed out that the recent decision of the members of the Consumer Healthcare Products Association, which represents the majority of manufacturers of OTC medicines for cough and colds in the United States, has been to voluntarily change the labels of these drugs to caution that they must not be used in children under 4 years of age. The American Academy of Pediatrics have advised against using dextromethorphan as well as codeine for treating any type of cough in the pediatric population, because no well-controlled scientific studies were found that support the efficacy and safety of these central acting drugs as anti-tussives in children. Indications for their use in children have not yet been established [60]. In 2009 the Medicines and Healthcare products Regulatory Agency (MHRA) in the UK has discouraged the use of cough and cold remedies containing certain agents and has indicated that they should no longer be used in children under 6 due to an unfavorable risk-benefits ratio. For older children (6 to 12), pharmacological treatment of cough and colds is only recommended if basic principles of best care have failed. The products affected by this warning also included anti-tussives (dextromethorphan and pholcodine).

In January 2006 the American College of Chest Physician (ACCP) published the Evidence-Based Clinical Practice Guidelines on Diagnosis and Management of Cough. These guidelines recommend peripheral cough suppressant such as levodropropizine in adult patients with cough due to acute or chronic bronchitis for the short-term symptomatic relief of coughing [61].

In summary, acute and sub acute cough are very frequent and most episodes have a benign, self-limited course. A careful history and clinical examination is required to identify the occasional severe underlying condition that can present with a cough of acute or sub acute onset.

When the therapeutic intervention aimed at removing the underlying cause is unsuccessful, an early empiric symptomatic treatment of acute or sub-acute cough with anti-tussive agents is often needed in order to improve quality of life, restore physical and social and hopefully avoid the development of persistent cough with deterioration in the quality of life [56,57]. As far as the level of benefit is concerned, the effects of peripherally acting anti-tussives, such as levodropropizine and moguisteine, compare favorably with centrally-acting drugs, based on the evidence from clinical trials and according to the available clinical practice guidelines [47-49,51-55,61]. Thus, peripheral anti-tussive drugs have been recommended for the treatment of acute and sub-acute cough, both in children and adults

#### Chronic cough

The objective of managing chronic or persistent cough is to address its cause. Several prospective studies have shown that adequate treatment of specific aetiologies of chronic cough is effective in the vast majority of cases [3]. However, under certain circumstances, the cause of cough is not treatable, even if it is known (e.g., endobronchial lung cancer or pulmonary fibrosis). In such situations, a non-specific (symptomatic) anti-cough therapy for symptomatic relief seems appropriate. Unfortunately, currently available cough suppressant drugs are often inadequate because of their limited efficacy, intolerable side effects, or both [62]. Despite evidence-based medicine supporting the use of empirical therapy in adults with chronic cough, [5,19,63] there is no such evidence in children [64,65]. In fact, guidelines for chronic cough from both the U.S., and Europe, recommend the empiric use of inhaled corticosteroids for adult patients when treating cough due to asthma, proton pump inhibitors when the cough is associated with GERD, and first-generation anti-histamines when it is subsequent to an the upper airway syndrome. In children, however, this approach is neither recommended by the U.S. guidelines nor the Australian *position paper *in light of the fact that evidence for the use of these empirical therapies is lacking in younger age groups and that medications cause significant side effects, especially when used at high doses and for prolonged periods of time [66-72]. While GERD and cough syndrome of the upper airways might not be as common in children where protracted bacterial bronchitis is more frequent as well as spontaneous resolution of cough with no apparent link between resolution and the treatment [70].

Therefore, it is not surprising that in adults, first-generation anti-histamines appear to be effective [31] which, however, is questionable in children [32].

Health professionals often recommend the use of Self-prescribe OTC preparations for the initial treatment of cough, although there is little evidence for their effectiveness.

A correct explanation of the natural history of non-specific cough, such as cough associated with single or multiple viral infections that resolve naturally without pharmacological intervention will probably help patients understand the problem and the true extent of their cough. This should in turn lead to a reduction in the unnecessary use of drug treatments or alleged remedies. However, if there is an indication for symptomatic treatment of a cough, e.g. in situations in which causal treatment of a cough is not effective (e.g., advanced lung cancer or interstitial lung disorders) as well as in patients with chronic bronchitis where short-term control of symptoms is necessary should be used only drugs which have documented clinical efficacy and/or guideline recommendations. Among these peripherally acting anti-tussives, such as levodropropizine and moguisteine, have been recommended when symptomatic relief of a chronic or persistent cough is indicated. This recommendation is based on published evidence from clinical trials and subsequent clinical practice guidelines, where these drugs have shown clinical efficacy with a favourable benefit/risk profile, especially in the treatment of cough due to chronic bronchitis [28,48-50,61].

## Conclusions

Cough is one of the most common symptom that results in medical consultations [73] and is the most frequent complaint of patients seeking advice from practicing pulmonary physicians, accounting for up to 40% of the practice outpatient care activity [12,74]. Coughing is an important defensive reflex that enhances clearance of secretions and particulate matter from the airways and protects from aspiration of foreign materials occurring as a consequence of aspiration or inhalation of particulate matter, pathogens, accumulated secretions, postnasal drip, inflammation, and associated inflammatory mediators. Under normal conditions, cough plays an important protective role in the airways and lung parenchyma, but in some conditions coughing may become excessive and nonproductive, and is troublesome and potentially harmful to the airway mucosa.

The potential benefits of treating cough early could be in preventing the vicious cycle of cough perpetuating cough [56]. The treatment of cough often requires symptom related approaches. Empiric treatment with anti-tussive agents is particularly needed when associated with deterioration in the quality of life [57]. Available drugs for symptomatic treatment of cough include both peripheral and central products. Recently, both the FDA and MHRA recommended against the use of OTC products for coughs and colds, including central anti-tussives, in infants and young children. Furthermore, the American Academy of Paediatrics have advised against using dextromethorphan as well as codeine for treating any type of cough in the paediatric population [60]. The American College of Chest Physicians (ACCP) issued their evidence based "Guidelines on Cough" in 2006, which state that anti-tussive drugs related with therapy of acute or chronic bronchitis showing the highest level of benefit were levodropropizine and moguisteine, that act through a peripheral mechanism, while the central antitussive drugs such as codeine and dextromethorphan showed a lower level of benefit.

## Competing interests

- Publication of this article was supported by Dompè SPA, Italy (unrestricted grant). 

- G. De Danieli, R. Balsamo and L. Lanata are employees of Dompè SPA, Italy.  

- F. De Blasio, Johann C. Virchow, M. Polverino, A. Zanasi, P.Behrakis and G.Kilinc have received an honorarium for participating in an advisory board for Dompè SPA.  

## Authors' contributions

FDB carried out the conception of study and set up the task group of the authors. All authors carried out the literature review. All authors carried out the draft paper. All authors read and approved the final manuscript.
